# On Social and Cognitive Influences: Relating Adolescent Networks, Generalized Expectancies, and Adolescent Smoking

**DOI:** 10.1371/journal.pone.0115668

**Published:** 2014-12-23

**Authors:** Cynthia M. Lakon, John R. Hipp

**Affiliations:** 1 Department of Population Health and Disease Prevention, Program in Public Health, University of California Irvine, Irvine, California, United States of America; 2 Departments of Criminology Law and Society and Sociology, University of California Irvine, Irvine, California, United States of America; University of Bristol, United Kingdom

## Abstract

We examine the moderating role of friendship and school network characteristics in relationships between 1) youths’ friends smoking behavior and youths’ own generalized expectancies regarding risk and future orientation and 2) generalized expectancies of youths’ friends and youths’ own generalized expectancies. We then relate these constructs to smoking. Using a longitudinal sample from the National Longitudinal Study of Adolescent Health (N = 15,142), the relationship between friends’ generalized expectancies and youths’ expectancies is stronger for those more central in the network, with more reachability, or stronger network ties, and weaker for those with denser friendship networks. Risk expectancies exhibited an inverted U shaped relationship with smoking at the next time point, whereas future orientation expectancies displayed a nonlinear accelerating negative relationship. There was also a feedback effect in which smoking behavior led to higher risk expectancies and lower future orientation expectancies in instrumental variable analyses.

## Introduction

Numerous studies have examined the relationship between social network characteristics and various health and risk behaviors across populations [1]–[3]. A voluminous and growing body of literature has yielded keen insights into pathways linking adolescent social networks and risk behaviors including smoking; notably studies utilizing Stochastic Actor-Based Models [4], [5] have elucidated linkages between degree based, triadic, and other network effects and both friendship tie choice and adolescent smoking, for example [6]–[9]. Other studies yet have taken alternative approaches to gaining insight into pathways linking adolescent friendship networks and smoking behavior, finding that emotional support linked network characteristics and adolescent smoking behavior [10]. While such studies have yielded valuable insights, more theoretical work is necessary to further delineate the complex linkages between adolescent networks and smoking behavior, particularly research explicitly linking adolescent network characteristics with cognitive processes which might impact adolescent smoking.

As such, the present study considers whether adolescents’ cognitive expectancies, in part developed within their peer networks, have consequences for subsequent smoking behavior. Expectancies are learned expectations that some reinforcement or grouping of reinforcements will occur in any given situation or across situations [11]. Foundational theories of health behavior emphasize expectancy constructs in learning, attitude formation, and engagement in health behavior [12], [13]. Social Cognitive Theory [13] posits that the social environment, cognition, and behavior, are in constant interaction as humans learn to adapt to their environments via numerous processes including the formation of expectations and social influences. For adolescents, peers are important role models, and are sources of information, validation, and social influence, all of which affect youths’ decision making about risk behaviors such as smoking. The positive relationship between peer influence and adolescent smoking is well documented [14], [15]. Studies conceptualize peer influence in relation to adolescent smoking in various ways, including the number of friends youth have who smoke [16].

**Figure 1:**
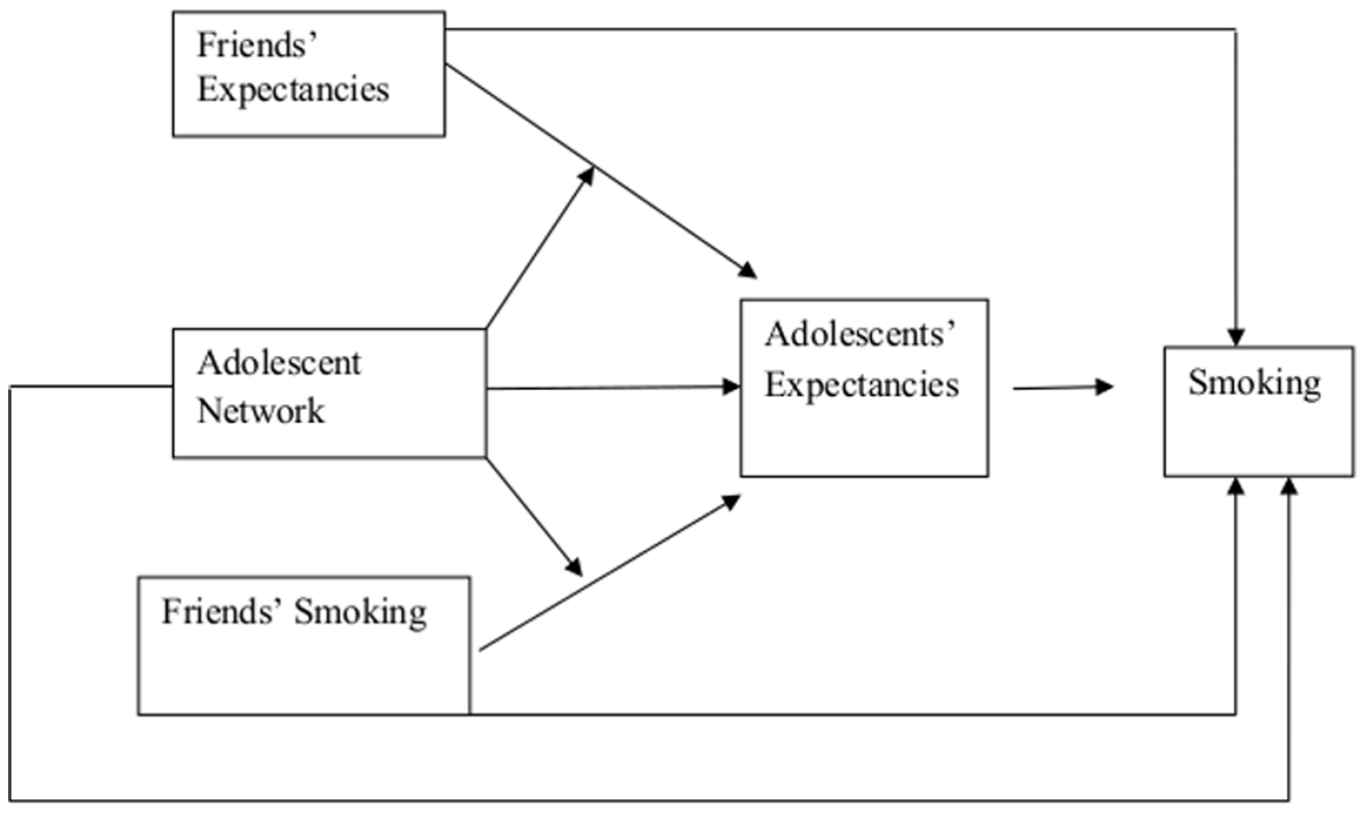
A conceptual model depicting pathways linking adolescent network characteristics, expectancies, and smoking.

Premised upon intuition from Social Cognitive Theory, this study considers that youths’ social networks might play a moderating role in shaping adolescents’ expectancies through learned associations made via social interactions with peers and through the structure of these networks. Thus, youths’ expectancies may be affected by those held by their friends given the propensity towards similarity among friends on multiple dimensions during adolescence [17], [18]. Youths’ expectancies are likely a product of the expectancies of one’s network members along with the network structure. We posit a conceptual model (see Fig. 1) and employ an accompanying two-stage modeling strategy which implies that expectancies are key constructs along pathways through which adolescent network characteristics impact smoking. We examine whether characteristics of adolescents’ personal networks of friends and the larger school wide network shape youths’ own expectancies, and also test whether youths’ own expectancies relate to their smoking behavior. Specifically, our model delineates the following direct and moderated pathways linking characteristics of adolescent networks, youths’ own expectancies, the expectancies held by youths’ friends, their friends’ smoking behavior, and youths’ own smoking behavior. The direct relationships under study relate: 1) characteristics of adolescents’ networks and both friends’ expectancies and friends’ smoking behavior to adolescents’ own expectancies; 2) characteristics of adolescents’ networks, friends’ smoking behavior, and their friends’ expectancies to youths’ own smoking behavior, and 3) youth’s own expectancies to their smoking behavior. Moderated pathways examine whether adolescent network characteristics moderate the relationship between both friends’ expectancies and friends’ smoking behavior and youths’ own expectancies. Another pathway examines whether one type of expectancy under study, risk perception, has a nonlinear relationship with adolescent smoking. In what follows, we describe the rationale for the expectancies, network characteristics and pathways under study. We are not aware of studies conceptualizing both direct and moderated pathways linking adolescent networks, their own expectancies, their friends’ expectancies and friends’ smoking behavior, with their own smoking behavior.

### Generalized Expectancies

The role of expectancies in adolescent drug use is well documented [19]–[21]. Studies have found that smoking related expectancies–specific beliefs regarding the potential consequences of smoking-are associated with adolescent smoking [22]. Past research also finds a strong relationship between general health related expectancies and adolescent smoking [23]. While expectancies have been defined in various ways, the *generalized expectancy* is of interest in this study. Such expectancies arise from a specific set of circumstances and generalize to a sequence of situations which are perceived as similar, which then forms a generalized expectancy for this grouping of related events or behaviors [24]. Theories of adolescent risk behavior incorporate generalized expectancies, notably Problem-Behavior Theory [25] as adolescents’ perceived chances of future life events occurring. Past studies have examined the relationship between adolescents’ perceived life chances and adolescent risk behaviors [26]–[31].

While research has demonstrated the importance of generalized expectancies for adolescent problem behaviors, a recent review of the adolescent decision-making literature [32] emphasized the need for research which *contextualizes* decision-making processes within youths’ social contexts. We follow this call by studying how youths’ networks relate to their generalized expectancies. We focus on two generalized expectancy constructs salient to adolescent smoking: 1) risk perceptions and 2) future time perspective.

Risk perceptions are subjective judgments about uncertain events and other potential hazards, and come into play in foundational theoretical perspectives describing health behavior [12], [33]. During adolescence, youth make decisions regarding risk; this involves processing subjective probability judgments about uncertain events occurring in their lives [34].

Interestingly, studies indicate a lack of clarity regarding the nature and direction of the relationship between risk perception and adolescent smoking. Rational choice models would predict a negative relationship between risk perception and adolescent risk behavior. Indeed, some studies find such a relationship regarding smoking [35]. Likewise, longitudinal studies indicate that adolescents perceiving lower risks of deleterious health consequences from smoking are more likely to begin smoking [36]. However, some research indicates that adolescents who perceive more risks engage in *more* risk behavior [37]–[39]. One possibility for these mixed findings is that a reciprocal relationship between risk perception and smoking exists; as Gerrard et al. [39] found a reciprocal relationship between perceptions of vulnerability and adolescent risk behavior including smoking. Another possibility is that the relationship between risk perceptions and smoking is non-linear, as there may be a threshold point beyond which high levels of risk perception actually reduce smoking behavior. Gerrard et al. [39] suggest that individuals who engage in a risk behavior and do not experience negative consequences from doing so may continue the behavior based on the “absent/exempt” perspective of Weinstein [40]. Moreover, these researchers indicate that the relationship between perceptions of vulnerability and risk behavior is initially linear, at some point plateaus and then eventually decreases, and that an inverted-U shaped relationship between perceived vulnerability and risk behavior might be found in studies which utilize adolescent samples spanning the adolescent age range, thus capturing the trajectory of engagement in risk behavior. Given the lack of clarity regarding how risk perception relates to adolescent smoking, our study examines this as a non-linear relationship by testing the quadratic effect of risk perception.

The second type of generalized expectancy under study is having a temporal orientation towards the future, which is based on evidence that humans perceive time in past, present, and future reference frames [41]. Whether adolescents engage in risk behavior is related to their temporal orientations towards their futures [42]. Present time perspective, an orientation towards present events, is positively related to adolescent smoking and other substance use [43]. Future time perspective is an orientation towards future events [44]. Adolescents employing a future time perspective are likely able to conceptualize long term goals and are aware of the long term consequences of their actions. One study found that adolescents with a future time perspective (i.e., those who perceived higher chances of attending college or higher expectations of living to age 35) smoked fewer cigarettes in young adulthood [30]. In another study, adolescents reporting lower chances of living to age 35 were more likely to smoke or use illicit substances, and had higher suicidal ideation and attempt [31].

### Adolescent Network Characteristics

Following the insights of Social Control Theory [45], we posit that adolescents who are socially integrated in their schools will express different generalized expectancies and will be less likely to smoke compared to their less integrated counterparts. Youth who are less tightly bound within the social structure of the school may be more likely to smoke. Indeed, studies generally find that less socially integrated youth (e.g., isolates and those who act as liaisons or bridges between otherwise disconnected groups) are more likely to smoke [46]–[48], although one study of young adolescents in sixteen predominantly Hispanic/Latino Southern California middle schools found that popularity positively relates to susceptibility to smoking [49]. We expect that our general population sample, which spans the adolescent age range, will exhibit results consistent with the general pattern of studies in this area.

We therefore examine whether network characteristics capturing social integration in youths’ networks are related to youths’ generalized expectancies, which might then relate to smoking behavior. We are particularly interested in how certain network characteristics might moderate the relationship between friends’ expectancies or friends’ smoking behavior and youths’ own expectancies. We measure *social integration* in networks using: (1) degree centrality [50] which reflects prestige in a network, (2) reachability or “reach”, which is the number of youth an adolescent may know via direct and indirect ties in the network [51] (3) the density or extent to which adolescents’ friends know one another within their personal network, and (4) the strength of adolescents’ ties to those in their friendship networks. We also examine a network characteristic indicating *less social integration* in school based networks: the number of nominated friends outside the school. Next, we draw on the adolescent social network and smoking literature to provide theoretical intuition for why these network characteristics are important for the generalized expectancies under study and smoking.

### Linking Friendship Network Characteristics, Generalized Expectancies & Smoking

Popular youth are directly connected to many others in their network and tend to set and reflect normative trends with respect to smoking in their setting [49]. These youth are therefore in a structural position to be strongly exposed to peer influences condoned by the mainstream in their setting. Consequently, the generalized expectancies regarding risk and future orientation of popular youth and their smoking behavior are likely to align with those held by the popular mainstream, be they risk protective or risk promotive for smoking. Therefore, it is likely that the generalized expectancies of popular youth will more closely mirror the expectancies of their friends than will be the case for less popular youth.

Another salient network characteristic for the generalized expectancies under study and smoking behavior is reachability or “reach.” Reach has implications for diffusion in a network [52], including the transmission of peer influences and information [53]. Youth with high reach are likely exposed to diverse informational inputs and a broad range of peer influences given their high level of integration. Thus, reach may affect youth’s generalized expectancies towards risk and future orientation given that youth with high reach may learn from social interactions with diverse network members which generalize across situations. This raises the question of whether youth with high levels of reach are more likely to adopt their friends’ generalized expectancies, who may hold a varied set of generalized expectancies. In contrast, socially isolated adolescents with a lower reach will have fewer opportunities for exposure to diverse informational inputs, which may circumscribe learning in their environment which will in turn affect their generalized expectancies and smoking.

The density of personal network ties may impact adolescents’ exposure to friends’ generalized expectancies and smoking behavior. Densely connected network ties are thought to increase regulatory social influences exerted in a network (Laumann, 1973; Krohn, 1986). Thus, dense network ties may align youths’ expectancies and smoking behavior with predominate expectancies and smoking behavior in the network. In one study, Haynie [54] found that network density moderated the delinquency-peer association, with dense networks containing stronger delinquency-peer associations than those that are less dense. Other research indicated that adolescents with denser networks had lower odds of recent smoking [48].

Also important for adolescents’ generalized expectancies and smoking is the strength of their network ties. Granovetter conceptualized tie strength as the sum of the time investment, emotional intensity, intimacy, and services exchanged within a social tie [55]. These strong and sometimes longstanding ties may be conduits of influence in adolescent friendships, and as such youth may hold similar generalized expectancies and smoking behaviors. Indeed, research shows that close friends display homogeneous smoking behavior [56].

Adolescents’ friendship ties outside school may capture inputs from a potentially important set of friends regarding both generalized expectancies and smoking. It is unclear what kind of impact these friends have on youths’ generalized expectancies and smoking, however, interacting with these youth likely provides diverse opportunities for learning that may differ from those youth are exposed to with school friends. Having friends outside of school has been positively associated with adolescent smoking [48]. In addition, in schools in which students nominate numerous friends outside of school, the school and network boundary cannot be equated, perhaps lessening the prominence of any characteristic within the school [57].

School networks comprise all students in a school and the relationships among them. We are not aware of any studies examining how characteristics of school networks relate to youths’ generalized expectancies. We examine three school level network characteristics which are likely salient to generalized expectancies and smoking. First, we consider size, or the number of adolescents in a school and a geographically proximal “sister” junior or senior high school. Larger schools allow for more heterogeneous expectancies and smoking behavior. In larger school networks, there is a higher probability of individuals holding diverse expectancies and engaging in a diversity of behaviors due to a larger number of people, providing a potentially broader range of influences and informational inputs to which these individuals are likely exposed. Second, we examine the density of ties or extent to which adolescents know one another in their schools. School level density captures the possible regulatory influence of the school network structure on the generalized expectancies and smoking, with denser networks exerting more pressure to conform to predominant peer influences in the school environment. Third, clustering of youth within their networks is the extent to which youth are highly and mutually connected in sub-areas of a network. Clustering may act to intensify and confine peer influences and informational inputs within densely connected regions of a network, which typically are areas of high homogeneity on beliefs and behaviors maintained by strong peer influences. Those within clusters may hold similar generalized expectancies and smoking behavior given their similar exposure to peer influences and other informational inputs.

### Current Study

The current study examines direct and moderated pathways linking adolescent’s personal and school network characteristics, their own generalized expectancies towards risk and future orientation, their friends’ expectancies and smoking behavior, and their own smoking in a large and nationally representative sample. Given the large and heterogeneous sample under study, we expect that personal network measures indicating social integration will negatively relate to smoking, while measures indicating less integration will positively relate to smoking. We expect that the network characteristics will moderate the relationships between both friends’ smoking behavior and expectancies and youths’ own generalized expectancies. We also expect that the network characteristics reflecting social integration will be positively related to future orientation expectancy, and that having ties outside of school will be negatively related to future orientation expectancy. We expect that risk expectancy will be nonlinearly related to smoking and future orientation expectancy will be negatively related to smoking. We hypothesize that friends’ smoking will be positively related to adolescent smoking.

## Methods

### Participants

Data come from the In-School and first In-Home waves of the National Longitudinal Study of Adolescent Health [58]. The In-School survey was administered to students in a nationally representative sample of schools in grades 7 through 12 from September 1994 through April 1995. The first In-Home survey was administered to a random sample of these students approximately six months later. After eliminating the 5,239 students who did not answer the question about smoking behavior at wave 2 or were not identified to a school in wave 1, the study sample was 15,142 students attending 133 schools. This study approved by the University of California, Irvine Institutional Review Board. Add Health participants provided written informed consent for participation in all aspects of Add Health in accordance with the University of North Carolina School of Public Health Institutional Review Board guidelines that are based on the Code of Federal Regulations on the Protection of Human Subjects 45CFR46: http://www.hhs.gov/ohrp/humansubjects/guidance/45cfr46.html. Written informed consent was given by participants (or next of kin/caregiver) for their answers to be used in this study.

### Measures

There are three dependent variables. Smoking behavior at wave 2 was measured as the number of cigarettes smoked in the last month. We also included as a covariate smoking behavior at wave 1, based on an ordinally scaled question asking respondents how often they smoked cigarettes in the last year (0 = never; 1 = once or twice; 2 = once a month or less; 3 = 2 or 3 days a month; 4 = once or twice a week; 5 = 3 to 5 days a week; 6 = nearly every day).

### Generalized Expectancies

We created two generalized expectancy scales at time 1 as dependent variables: 1) future orientation expectancy and 2) risk expectancy. We specified a two factor confirmatory factor analysis (CFA) model and computed factor scores from this model. The model fit was excellent for this CFA: although the chi square was significant (χ^2^ = 58.8 on 3 *df* at wave 1), this is largely due to the large sample size. Whereas root mean squared error of approximation (RMSEA) values less than .05 are considered satisfactory, our value of .036 suggests excellent fit. Likewise, whereas values for the Comparative Fit Index (CFI) or the Tucker-Lewis index greater than .9 or .95 are considered satisfactory, our values of .99 again suggest excellent fit.

The generalized expectancy indicators comprising the scales asked respondents: “What do you think are the chances that each of the following things will happen to you?”(1 = no chance, 2 = some chance, 3 = about 50–50, 4 = pretty likely, 5 = it will happen). The measure of *risk expectancy* used the following questions: 1) you will be killed by age 21; 2) you will live to age 35; and 3) you will get HIV or AIDS. This measure represents a perceived susceptibility dimension of risk relating to disease and mortality, see [59]. Perceived susceptibility constructs have played a key role over the last few decades in foundational theories of health behavior such as the Health Belief Model [33], [60]. The measure of *future orientation expectancy* combined three questions: 1) you will be married by age 25; 2) you will graduate from college; and 3) you will live to age 35. This construct taps into youths’ cognitive time frame and whether it is oriented towards the future.

To assess construct (i.e., both convergent and divergent) validity of the generalized expectancies, we correlated the both with other measures, including: 1) *danger due to a dare in the last 6 months* was correlated .25 with risk and −.23 with future orientation expectancies*; try do to school work well* was correlated −.21 and .23, respectively; and a measure of *getting drunk in the last 12 months* was correlated .25 and −.27, respectively. These correlations are in the expected direction with future orientation expectancy. In ancillary regression analyses, we found an increasing nonlinear relationship between risk expectancies and these three measures, consistent with studies indicating a positive relationship between risk perceptions and engagement in risk behavior. We also assessed whether *risk expectancies* simply captured perceived risk due to living in a dangerous neighborhood: the correlation was effectively zero with the *poverty rate*, *unemployment rate*, or *median income of the neighborhood*, and the *county violent crime rate*. The correlation between *future orientation expectancies* and the *percentage with at least a bachelor’s degree in the neighborhood* was only .07, suggesting that this measure does not simply capture perceptions of neighborhood context.

### Social Network Measures

A network elicitation item asked respondents to nominate up to 5 male and 5 female friends, these are respondents’ *alters.* Respondents could name persons in their school, at a geographically proximal “sister” junior high school, and friends outside school. Personal networks are local friendship network subsets of larger school networks. School networks comprised *all* students in a school and relationships among them.

*Bonacich Centrality* reflects the centrality or prestige of an individual in a network, and is weighted by the centrality of his or her friends; we set β to a small positive value (.1) to reflect positive prestige and to more heavily weight the local structure of the network [50]. *Reach* is the number of persons an adolescent can reach directly or indirectly in the network. *Personal network density* computes the number of ties in a personal network divided by the number of possible ties. This proportion is undefined in networks with one or no ties and is given missing values; therefore as a methodological solution, we include an indicator for *near-isolates* ( = 1 if one or no ties). *Average tie strength* was created by constructing the strength of each tie named by an adolescent based on a sum of whether the adolescent reported engaging (yes/no) in the following activities with an alter in the last seven days: a) went to their house; b) met after school to hang out; c) spent time last weekend; d) talked on the telephone, and then computed the sum over all alters in the personal network. Lastly, *ties outside of school* measured the number of youth respondents named as friends who were not in their schools. All measures were created using Jim Moody’s SPAN program [61].

At the school network level, *school size* captures the number of youth in a school. School *network density* is the number of relationship ties in the school divided by the total possible ties that could exist. The *clustering coefficient* measures the average density in the personal networks within a school.

We constructed measures of friends’ smoking behavior and friends’ expectancies. We measure friends’ smoking behavior as the average smoking behavior of youth’s friends in their personal networks, which is consistent with measures in past studies [16]. We also examine the *average expectancy* score for one’s friends for both *risk* and *future orientation*, respectively.

We included demographic measures that relate to smoking and generalized expectancies. Gender was coded as *female*, (0 = *male, 1 = female)*. *Grade level* is coded continuously, ranging from 7 to 12. Race/ethnicity was coded as four dummy variables (*Black*, *Asian*, *Latino*, and *Other Race*, where *White* was the reference category). A dummy variable indicates whether the adolescent is an *immigrant*. We computed *parental support* as the mean of two questions regarding support from mother or father: “How much do you think she [he] cares about you?” (on a 5-point Likert scale ranging from “not at all” to “very much”), and *mother’s education* (1 = less than eighth grade; 2 = more than eighth grade but not high school graduate; 3 = high school graduate; 4 = some college; 5 = graduated from college; 6 = beyond 4 year degree).

We also account for the school composition. The average level of youths’ mother’s education in the school captures school socio-economic status. Racial/ethnic composition is measured by percentage of Blacks, Asians, and Latinos. We constructed measures of average smoking behavior, average risk expectancies, and average future orientation expectancies at the first time point.

We accounted for the modest amount of missing data with a multiple imputation strategy. The largest level of missing data was for mother’s education at 14%; however, on average the variables had just 2.7% missing values. We randomly imputed 50 datasets using the ICE command in Stata, which uses switching regression, an iterative multivariable regression technique. The results were combined using Rubin’s technique to correct the standard errors [62]. This requires the less stringent assumption of missing at random, rather than the stronger assumption of missing completely at random required of listwise deletion [62].

### Analysis strategy

Given the nesting of students in schools, we estimated multilevel linear models for three separate outcomes: the factor score of *risk expectancies*, the factor score of *future orientation expectancies*, and the *smoking* measure. Thus, we estimate the following level one equation:
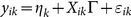
(0.1)where y*_ik_* is the outcome measure (for example, *future orientation expectancies*), reported by the *i*-th respondent of *I* respondents in the *k*-th school, η*_k_* is the latent variable of the outcome measure in the school (for example, *future orientation expectancies*), X*_ik_* is a matrix of exogenous predictors with values for each individual *i* in school *k*, Γ is a vector of the effects of these predictors on the outcome, and ε*_ik_* is a disturbance term.

The level two equation incorporates the school network characteristics, and is:
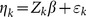
(0.2)where η*_k_* represents the overall generalized expectancies in school *k*, Z represents a matrix of school-level variables, β is a vector of the effects of these measures on the outcome, and ε*_k_* is a disturbance for school *k*. We also tested a quadratic term for each expectancy at time one, to allow for possible nonlinear relationships. In unconditional models, 21% of the variance in wave 2 smoking is between schools; likewise 18% and 12% of the variance in future orientation and risk expectancy is between schools, respectively.

We use temporality to account for possible feedback effects in the smoking equation: the outcome is measured at time 2 whereas the covariates are measured at time 1. For the two generalized expectancies equations, we accounted for possible feedback effects of the personal smoking covariate with instrumental variable estimation. We include two instrumental variables: parents’ smoking behavior; presence of cigarettes in the home noted by the interviewer. This predicted value of smoking from the first stage equation is then included in the expectancies equations. The Basmann and Sargan tests showed nonsignificant results, indicating that these instruments are uncorrelated with the error term. The first stage R^2^ of .20 is satisfactory, and the F-test value of 119.7 indicates that these are strong instruments [63].

We tested for moderation in the expectancies equations by creating interactions between friends’ risk expectancies, or future orientation expectancies, or smoking behavior, and the four network characteristics (Bonacich centrality, reach, personal network density, and average tie strength). There was no evidence of collinearity in these models, as variance inflation factors were all below 4. To further assess potential collinearity in the network measures we estimated ancillary models including one network measure at a time, and the results were effectively the same as those in the presented models.

## Results

In our sample, 51% were female, 24% were Black, 21% were Latino, 10% were Asian, and 6% were other race. Ten percent were immigrants. Adolescents had 1.5 ties outside the school, on average. Regarding smoking at wave 2, 75% did not smoke, 10% smoked one cigarette a day, about 2–3% fell into each of the categories 1–2 per day, 2–4 per day, and 4–6 per day, and 7.5% smoked more than half a pack a day. Table 1 presents the summary statistics for the study variables.

**Table 1:** Summary statistics of smoking, expectancies, network, and demographic measures.

Smoking	Mean	Std Dev
Smoking, wave 2	0.95	1.91
Smoking, wave 1	1.23	1.91
Average friends’ smoking behavior	0.73	1.14
***Expectancies***		
Future orientation expectancy	0.00	0.72
Average of friends’ future orientation expectancy	−0.02	0.48
Risk expectancy	0.00	0.24
Average of friends’ risk expectancy	0.00	0.20
***Personal network measures***		
Bonacich centrality	0.78	0.62
Reach	542.85	480.78
Density of personal network	0.31	0.15
Average tie strength	2.41	1.90
Number of ties outside school	1.48	2.00
Near-isolates	0.37	0.48
***School network measures***		
School size	936.82	552.34
School network density	0.42	0.10
School network clustering coefficient X 1000	0.43	3.74
***Demographic measures***		
Female	51.0%	
Grade	9.61	1.60
Black	25.2%	
Latino	20.7%	
Asian	9.7%	
Other race	5.7%	
Immigrant	10.1%	
Mother’s education	4.75	2.14
Parental support	4.68	0.67
***School demographic measures***		
Average smoking behavior	1.23	0.46
Average mother’s education	4.75	0.61
Average future orientation expectancy	0.00	0.12
Average risk expectancy	0.00	0.04
Percent Black	25.2%	22.4%
Percent Latino	20.7%	18.5%
Percent Asian	9.7%	11.6%
Note: 15,142 students in 133 schools		

### Generalized expectancies as outcome measure

Focusing on equations in which expectancies are outcomes, in equation 1 of Table 2, we find the expected positive relationship between average of friends’ future orientation expectancies and adolescent’s own future orientation expectancies (β = .095, *p*<.01), holding all else constant in this model. In equation 2 we observe a similar positive relationship between the average risk expectancies of one’s friends and oneself (β = .041, p<.01). There is no evidence that friends’ smoking behavior relates to one’s own expectancies when accounting for the measures in the model. However, those who smoke more have lower future orientation expectancies (β = −.118, p<.01) and higher risk expectancies (β = .032, p<.01), implying a possible reciprocal relationship between smoking and expectancies that we will return to in the equations with the smoking outcome.

**Table 2:** Multilevel models predicting expectancies and smoking.

	Future orientationexpectancy, time 1	Risk expectancy,time 1	Smoking,time 2	Smoking,time 2
*Time 1 measures*	(1)	(2)	(3)	(4)
Future orientation expectancy			−0.093**	
			−(5.04)	
Future orientation expectancysquared			−0.059**	
			−(5.64)	
Average of friends’ futureorientation expectancy	0.095**		−0.020	
	(7.44)		−(1.01)	
Risk expectancy				0.144**
				(2.73)
Risk expectancy squared				−0.429**
				−(5.26)
Average of friends’ risk expectancy		0.041**		0.059
		(4.21)		(1.30)
Smoking behavior	−0.118**	0.032**	0.574**	0.577**
	−(3.41)	(2.69)	(96.49)	(97.06)
Average friends’ smoking behavior	0.005	−0.005	0.162**	0.164**
	(0.29)	−(0.86)	(16.03)	(16.38)
***Personal network measures***				
Bonacich centrality	0.081**	−0.004	−0.003	−0.009
	(4.73)	−(0.74)	−(0.13)	−(0.44)
Reach (/1000)	0.088**	−0.042**	−0.046	−0.047
	(3.93)	−(5.42)	−(1.34)	−(1.38)
Density of personal network	−0.026	−0.025	0.078	0.081
	−(0.53)	−(1.50)	(1.02)	(1.05)
Average tie strength	0.001	0.005*	0.012†	0.011†
	(0.21)	(2.29)	(1.92)	(1.80)
Number of ties outside school	0.007†	0.001	0.014*	0.013*
	(1.94)	(0.44)	(2.39)	(2.36)
Near-isolates	0.015	−0.005	0.043†	0.043†
	(1.02)	−(1.03)	(1.84)	(1.85)
***School network measures***				
School size/1000	−0.041†	0.037**	0.039	0.055
	−(1.67)	(4.33)	(1.05)	(1.48)
School network density	−0.051	−0.012	0.000	−0.020
	−(0.42)	−(0.27)	(0.00)	−(0.11)
School network clusteringcoefficient	1.845	−1.046†	−2.883	−3.324
	(1.11)	−(1.82)	−(1.10)	−(1.26)
***Demographic measures***				
Female	0.101**	−0.014**	−0.022	−0.026
	(7.75)	−(3.11)	−(1.05)	−(1.25)
Grade	0.007	0.001	0.044**	0.041**
	(1.49)	(0.44)	(5.50)	(5.15)
Black	−0.121**	0.010	−0.181**	−0.170**
	−(4.49)	(1.10)	−(5.59)	−(5.26)
Latino	−0.159**	0.039**	−0.141**	−0.134**
	−(8.34)	(5.99)	−(4.60)	−(4.38)
Asian	−0.028	0.024*	−0.065	−0.067
	−(1.05)	(2.64)	−(1.55)	−(1.59)
Other race	−0.049†	0.021*	0.018	0.014
	−(1.72)	(2.18)	(0.41)	(0.31)
Immigrant	0.096**	−0.010	0.158**	0.153**
	(3.81)	−(1.13)	(4.07)	(3.94)
Mother’s education	0.0346**	−0.0008	−0.0085	−0.0108*
	(10.39)	−(0.69)	−(1.63)	−(2.10)
Parental support	0.2333**	−0.0704**	0.0641**	0.0591**
	(14.26)	−(12.51)	(4.11)	(3.83)
***School demographic measures***				
Average smoking level	0.096**	−0.029*	−0.005	−0.009
	(2.84)	−(2.44)	−(0.14)	−(0.25)
Average mother’s education	0.062**	−0.005	−0.052†	−0.035
	(3.83)	−(0.94)	−(1.90)	−(1.44)
Percent Black	−0.016	−0.054**	−0.143*	−0.205**
	−(0.31)	−(3.01)	−(2.00)	−(3.02)
Percent Latino	0.222**	−0.125**	−0.284*	−0.348**
	(2.90)	−(4.60)	−(2.48)	−(2.98)
Percent Asian	−0.100	−0.027	−0.008	−0.031
	−(1.04)	−(0.77)	−(0.05)	−(0.22)
Average future orientation expectancy			0.254*	
			(2.12)	
Average risk expectancy				−0.722*
				−(2.12)
Intercept	−1.748**	0.386**	−0.414*	−0.408*
	−(12.55)	(7.85)	−(1.97)	−(1.99)

Note: ** p<.01; * p<.05; † p<.1. T-values in parentheses. 15,142 students in 133 schools. All predictors measured at time 1.

Among the network measures, adolescents with stronger personal network ties hold higher risk expectancies on average (β = .005, p<.05). Adolescents with greater reach in their networks have higher future orientation expectancies (β = .088, p<.01) and lower risk expectancies (β = −.042, p<.01). More central adolescents hold higher future orientation expectancies (β = .081, p<.01). Adolescents in larger schools hold higher risk expectancies, on average (β = .037, p<.01).

Among the demographic measures, females have higher future orientation expectancies and lower risk expectancies. Blacks and Latinos have lower future orientation expectancies compared to Whites (the reference category), whereas Latinos, Asians and other race adolescents have higher risk expectancies. Immigrants and those with highly educated mothers have higher future orientation expectancies, whereas those with more parental support hold higher future orientation expectancies and lower risk expectancies.

### Moderating role of the network for generalized expectancies as outcome measure

**Figure 2:**
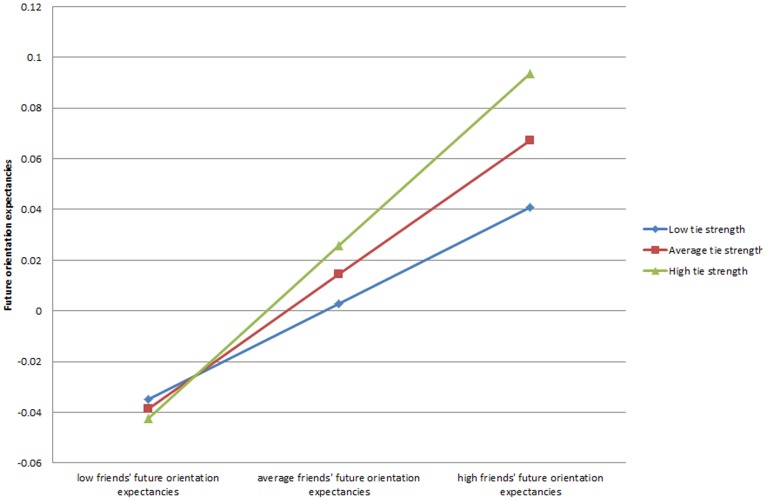
Future orientation expectancies predicted by interaction of average tie strength and friends’ future orientation expectancies.

We next examine the possible moderating role of the network characteristics in the relationship between friends’ generalized expectancies or smoking behavior and adolescents’ own future orientation and risk expectancies; these results are shown in Table 3. In model 1 we find strong evidence based on the interaction term that the strength of ties in one’s personal network enhances the strength of the relationship between the future orientation expectancies of an adolescent’s friends and their own expectancies (β = .016, *p*<.05). We plot these results in Fig. 2. The upward sloping lines indicate that higher levels of friends’ future orientation expectancies are associated with higher levels of future orientation expectancies by adolescents. In addition, although there are few differences in future orientation expectancies among adolescents whose friends have low levels of future orientation expectancies (the left side of Fig. 2), those whose friends have high levels of future orientation expectancies have higher levels themselves and particularly so when their personal network has high average tie strength (the right side of Fig. 2).

**Table 3:** Multilevel moderating models predicting expectancies.

	Future orientationexpectancies			
	(1)	(2)	(3)	(4)
Average of friends’ futureorientation expectancy	0.0681**	0.1579**	0.0683**	0.0727**
	(3.87)	(5.29)	(4.22)	(4.42)
Average tie strength	0.0060	0.0122	0.0077	0.0011
	(0.80)	(1.46)	(0.98)	(0.18)
Density of personal network	−0.0452	−0.0266	−0.0439	−0.0288
	−(0.92)	−(0.52)	−(0.87)	−(0.60)
Bonacich centrality	0.0656**	0.0528**	0.0552**	0.0788**
	(3.37)	(2.59)	(2.75)	(4.67)
Reach (/1000)	0.0833**	0.0776**	0.0833**	0.0936**
	(3.54)	(3.12)	(3.35)	(4.13)
Interaction of average tie strengthand friends’ expectancies	0.0158*			
	(2.50)			
Interaction of personal networkdensity and friends’ expectancies		−0.1537*		
		−(2.15)		
Interaction of centrality and friends’expectancies			0.0681**	
			(3.18)	
Interaction of reach (/1000) and friends’expectancies				0.0488*
				(2.09)
	**Risk expectancies**			
	**(5)**	**(6)**	**(7)**	**(8)**
Average of friends’ risk expectancy	0.0284*	0.0854**	0.0129	0.0232†
	(2.04)	(3.84)	(1.01)	(1.75)
Average tie strength	0.0036	0.0027	0.0033	0.0050*
	(1.42)	(0.95)	(1.22)	(2.29)
Density of personal network	−0.0208	−0.0321†	−0.0270	−0.0244
	−(1.23)	−(1.80)	−(1.54)	−(1.47)
Bonacich centrality	−0.0011	−0.0009	−0.0008	−0.0047
	−(0.17)	−(0.13)	−(0.11)	−(0.80)
Reach (/1000)	−0.0379**	−0.0416**	−0.0377**	−0.0403**
	−(4.70)	−(4.85)	−(4.44)	−(5.19)
Interaction of average tie strength andfriends’ expectancies	0.0071			
	(1.46)			
Interaction of personal networkdensity and friends’ expectancies		−0.1107*		
		−(2.09)		
Interaction of centrality andfriends’ expectancies			0.0636**	
			(3.65)	
Interaction of reach (/1000) andfriends’ expectancies				0.0383*
				(2.05)

Note: Models control for all variables shown in models 1 and 2 in Table 2.

Note: ** p<.01; * p<.05; † p<.1. T-values in parentheses. 15,142 students in 133 schools. All variables measured at time 1.

In contrast, in model 2 personal network density diminishes the relationship between friends’ future orientation expectancies and one’s own expectancies (β = −.154, *p*<.05). Thus, the densest personal networks weaken the relationship between the expectancies of one’s friends and oneself.

We find positive interaction effects between friends’ future orientation expectancies and those in central positions in the network (β = .068, *p*<.05; model 3) and those with high levels of reach (β = .049, *p*<.05; model 4). When we plot the interaction effect for centrality (not shown) it is very similar to Fig. 2 for the tie strength interaction. For the interaction between reach and friends’ future orientation expectancies, we find that those with higher reach have higher future orientation expectancies and that the relationship between friends’ expectancies and one’s own expectancies is strongest for those with high levels of reach (not shown).

**Figure 3:**
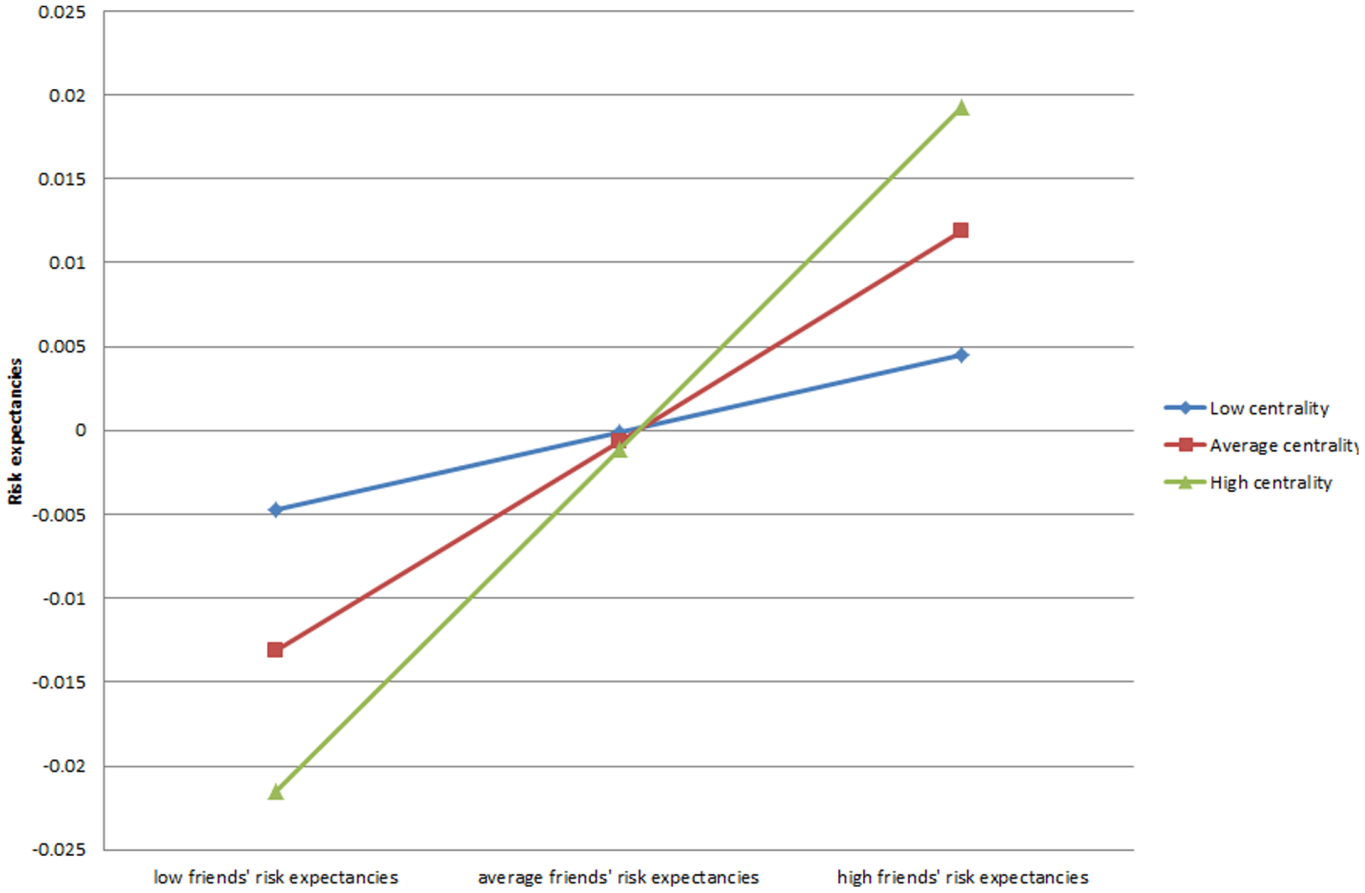
Risk expectancies predicted by interaction of centrality and friends’ risk expectancies.

Turning to the models with risk expectancies as the outcome, the interaction effect for average tie strength is not statistically significant. We see that denser personal networks weaken the relationship between the risk expectancies of one’s friends and oneself (β = −.111, p<.05). Plotting this interaction (not shown) revealed a relationship similar to that seen in Fig. 2 except that the top line in the graph is low density networks. We again find positive interaction effects for those who are more central (β = .064, p<.01), or with greater reach (β = .038, p<.05), (models 7 and 8). Plotting the result for the centrality interaction in Fig. 3 demonstrates that those with the highest centrality levels have lower risk expectancies when their friends have low risk expectancies (the left side of this figure) and matching high expectancies with their friends (the right side of the figure). Thus, their risk expectancies tend to be more similar with their friends’ risk expectancies. Finally, the interaction for reach indicates that whereas those with higher levels of reach have lower risk expectancies, this gap narrows when their friends have high risk expectancies (not shown).

We also estimated models in which we included interactions between the network measures and friends’ smoking behavior. In none of the models was the interaction statistically significant. Thus, whereas friends’ generalized expectancies are associated with an individual’s own expectancies through the network structure, friends’ smoking behaviors do not similarly impact an individual’s generalized expectancies.

### Smoking behavior at time 2 as outcome measure

**Figure 4:**
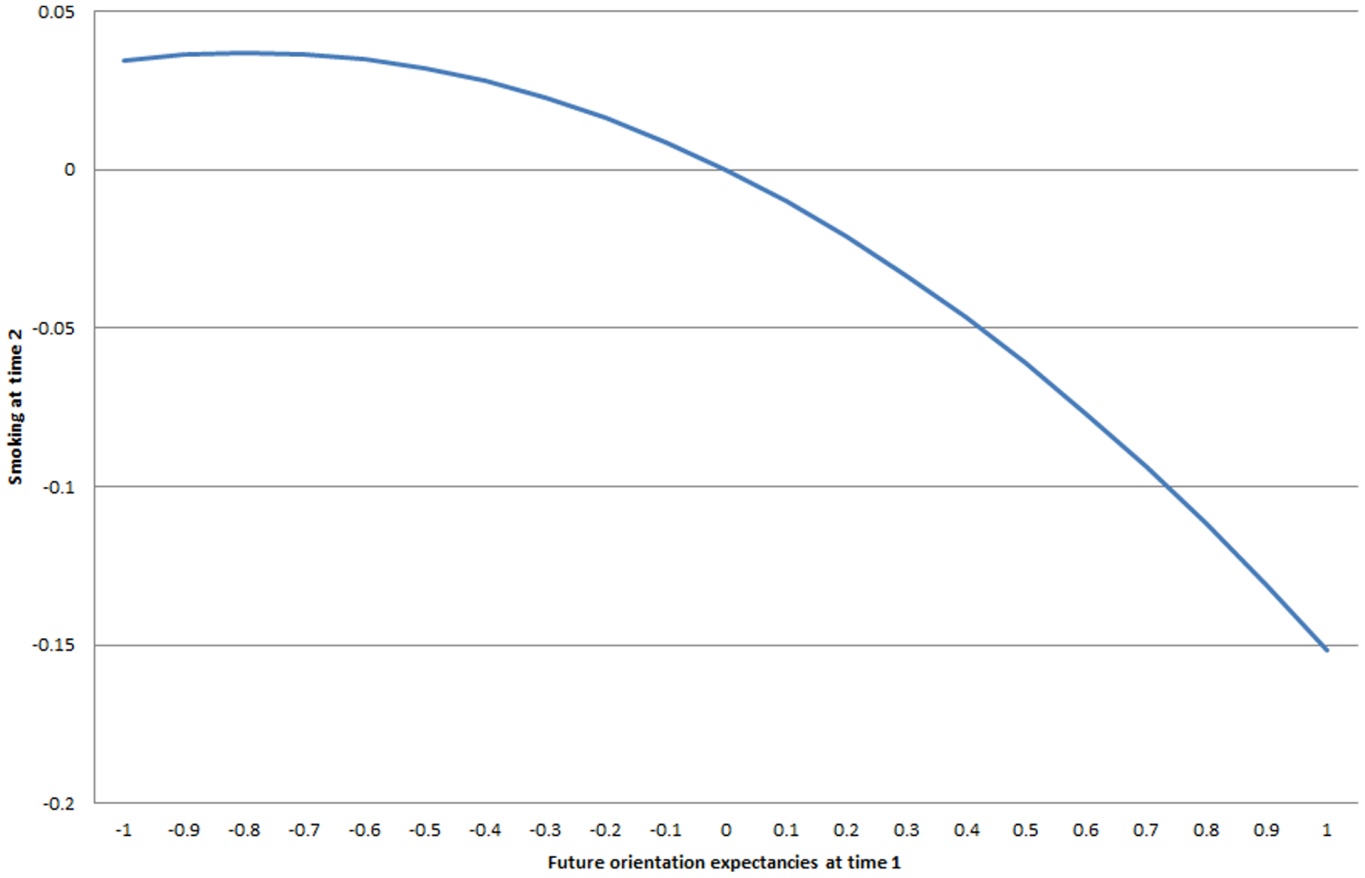
Effect of future orientation expectancy on smoking at next time point.

Turning to the models with smoking behavior at time 2 as the outcome, models 3 and 4 in Table 2 include respondents’ future orientation and risk expectancies respectively at time 1 as covariates. There are strong stasis effects, as smoking behavior at time 1 is positively associated with smoking behavior at time 2 in model 3 (β = .574, p<.01). There is also strong evidence that those with higher average smoking among their friends at time 1 engaged in more smoking behavior at time 2, even controlling for their level of smoking at time 1 (β = .162, p<.01; β = .164, p<.01). Nonetheless, respondents with higher levels of future orientation expectancies are much less likely to smoke at time 2 even controlling for prior smoking behavior (model 3): this is an accelerating nonlinear negative relationship as plotted in Fig. 4 (plotted from the 5^th^ to 95^th^ percentile of future orientation expectancies). Whereas the lower range of future orientation expectancies only have a modest negative relationship with smoking at time 2 (the left side of the figure), high levels have a sharp negative association with smoking (the right side of the figure). Thus, combining the results of models 1 and 3 implies a virtuous feedback cycle between future orientation expectancies and smoking: whereas model 1 in Table 2 found that lower levels of smoking at time 1 lead to higher future orientation expectancies (β = −.118), model 3 shows that higher future orientation expectancies result in reduced smoking at time 2 (Fig. 4).

**Figure 5:**
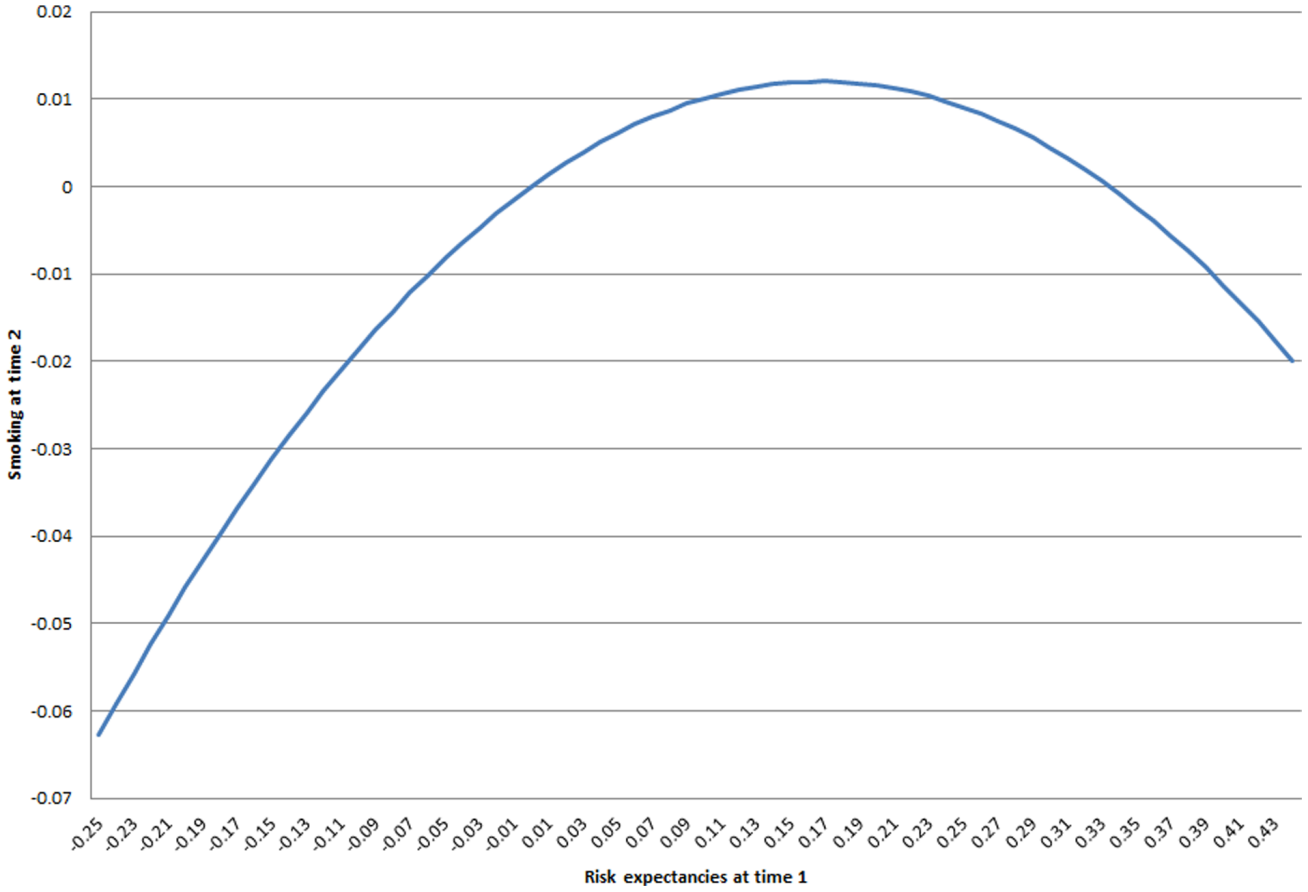
Effect of risk expectancy on smoking at next time point.

Turning to model 4, when plotting the quadratic relationship between risk expectancies at time 1 and smoking at time 2 Fig. 5 reveals a pronounced inverted-U relationship. Whereas increasing risk expectancies for those at the low end of the scale sharply increases smoking behavior at time 2 (the left side of the figure), increasing risk expectancies actually have a negative relationship with smoking at time 2 at the high end of the scale (the right side of the figure). There is no evidence that friends’ expectancies have an additional effect on smoking behavior at time 2.

Although the network measures were important for adolescents’ generalized expectancies, which then related to future smoking, there was little evidence that the network measures have direct effects on smoking (models 3 and 4 in Table 2). Only the measure of having more ties outside the school showed a direct positive relationship with smoking at time 2 (β = .013, p<.05; β = .014, p<.05).

Finally, the demographic measures relate to time 2 smoking, as expected. Blacks and Latinos smoke less than Whites at time 2, whereas those in a higher grade and immigrants smoke more. Although higher levels of mother’s education are associated with less smoking at time 2, parental support has an opposite relationship. There are school contextual effects: adolescents in schools with higher average future orientation expectancies and lower average risk expectancies at time 1 will smoke more at time 2. Notably, these unexpected results become non-significant if the school measures are excluded from the model, suggesting that these school-level expectancy results are only obtained when controlling for the school composition.

## Discussion

Our findings provide evidence that whereas generalized expectancies are related to smoking at the next time point, these expectancies are a product of the generalized expectancies of one’s network members along with the network structure. Our two-stage modeling strategy implies that expectancies are key constructs along pathways through which network measures relate to smoking. Adolescents more central in the network, or more well integrated based on reach, reported higher levels of future orientation expectancies, and these future orientation expectancies led to less smoking at the following time point. Those with less reach *and* stronger ties report greater risk expectancies; these risk expectancies showed a pronounced nonlinear relationship with future smoking as increases at the lower end of the scale were associated with more future smoking, but this relationship actually turned negative at the highest levels of risk expectancy. Overall, findings suggest that expectancies play a complex role in the relationship between characteristics of adolescents’ personal networks and their smoking behavior.

A novel contribution of this study was focusing on the relationship between adolescent networks and generalized expectancies, and the possible consequences for smoking. For instance, our interaction models indicate that those who are more central in the network are more likely to hold generalized expectancies in concert with those of their friends. Thus, highly central adolescents with friends who hold higher future orientation expectancies hold higher future orientation expectancies themselves. Likewise, highly central adolescents will tend to mirror their friends’ level of risk expectancies (see Fig. 4). A highly central adolescent with friends who are high in future orientation expectancies will therefore tend to have higher future orientation expectancies, and these higher future orientation expectancies are associated with less smoking over time.

Having high levels of reach also had implications for the two types of generalized expectancies studied here. For adolescents with high levels of reach, their own generalized expectancies are more likely to mirror those of their friends. Thus, those with high levels of reach and friends with high future orientation expectancies have higher future orientation expectancies, which are associated with less smoking at time 2. Reach likely captures the potential for information flow in the network, and our findings that individuals with greater reach may be more influenced by expectancies of *both* types is consistent with the idea that youth with a far reach are exposed to diverse informational inputs given that they have the potential for many and diverse friendships. This finding highlights some of the many functions network ties serve, including possibly as conduits for information and influence. The transmission of peer expectancies through networks may be particularly important for fostering adolescents’ expectancies.

It was also the case that those with stronger ties to their friends were more likely to hold higher future orientation expectancies if their friends held high future orientation expectancies, as shown in Fig. 2. These higher future orientation expectancies have an accelerating negative relationship with future smoking (even controlling for the level of smoking at the prior time point), suggesting that these strong ties play an important role in this relationship. The same pattern was not detected among those whose friends hold low levels of future orientation expectancies, nor was it detected in relation to the risk expectancies of friends.

Whereas the presence of strong ties in a personal network increase the similarity between the generalized expectancies of an adolescent and his or her friends, density operated differently. We instead detected a negative interaction effect, such that in personal networks with *low* levels of density and friends with higher levels of generalized expectancies, an adolescent will tend to report higher levels of generalized expectancies compared to those with personal networks with higher density. This pattern was found for both types of generalized expectancies. We had hypothesized that higher density would in fact have a positive moderating effect rather than this negative one. This one unexpected finding suggests a useful avenue for future research to explore why this might be the case.

Another key contribution of this study was in detecting a strong and reinforcing reciprocal relationship between future orientation expectancies and smoking. Prior research has not parsed apart these effects. On the one hand, adolescents with higher future orientation expectancies smoked less at the next time point, and this relationship was pronounced for those with very high levels of future orientation expectancies. On the other hand, we also detected a feedback effect in an instrumental variable analysis that accounted for these interdependent relationships: adolescents who smoked less expressed higher future orientation expectancies.

The reciprocal relationship detected between smoking and risk expectancies was more nuanced. Although smoking behavior linearly increased risk expectancies in an instrumental variable analysis, there was an inverted-U relationship between risk expectancies and smoking at the next time point. The positive relationship between risk expectancies and smoking for those in the lower range of risk expectancies becomes negative at the highest levels of risk expectancies. This change in the direction of the relationship may imply a threshold beyond which risk expectancies become threatening and the behavior less frequent. Gerrard and colleagues (1996) suggested a possible inverted-U relationship between perceptions of vulnerability and risk behavior in samples spanning the adolescent age range, given that risk behaviors are just developing earlier in adolescence and perceptions of vulnerability will therefore not increase indefinitely but instead level off and begin to decelerate, impacting the behavior. Perhaps the age range and heterogeneity of our sample aided in the detection of this relationship.

This study has some limitations of note. The networks were limited to up to 5 female and 5 male friends, which may not account for all of the youths’ closest friends. It is unclear how truncating the networks in this way may have affected the findings. Relatedly, we did not have full information about those outside school whom adolescents nominated. Third, it is unclear how students who were eliminated from the study sample because they did not answer the question about smoking behavior affected our study findings. Fourth, this study is limited by the secondary data source, which is most relevant for measuring the generalized expectancies and the parental variables under study.

Despite these limitations, our findings have notable implications for future research. First, our findings indicate a need for further examination of how adolescent network characteristics relate to these expectancies. Findings also suggest testing other specifications of nonlinear relationships between the expectancies and smoking, across various age ranges of adolescent samples. Moreover, findings suggest merit in the general theoretical strategy of testing the role of expectancies in the context of adolescent network characteristics for smoking. Findings also suggest that reciprocal relationships between expectancies and smoking should be studied in other adolescent populations. Future research is necessary to study the role of generalized expectancies in linking adolescent social networks to smoking behavior, to disentangle the nuanced linkages between adolescents’ friendship networks, generalized expectancies, and smoking. Our novel theoretical approach is a first step toward such future research.

Our findings also have practical implications for intervention programs targeting adolescents’ cognitions and smoking behavior. Our findings suggest targeting youth who are central in their networks or who have greater reach, to reinforce future orientation expectancies among youth in their personal network, which we find then related to less smoking. Our findings also suggest understanding when youth experience shifts in their risk expectancies and correspondingly when their smoking behavior starts to decelerate, to help youth make those shifts which correspond to decreasing smoking behavior earlier in time.

## Conclusions

In sum, our findings provide evidence that adolescents’ generalized expectancies regarding risk and future orientation act as key variables along theoretically nuanced pathways linking youths’ network characteristics and smoking, along with friends’ expectancies and smoking behavior in youths’ networks. Findings suggest that these networks and friends’ expectancies alike played a nuanced and multiplicative role in shaping youths’ generalized expectancies. As such, future theoretical work is necessary to more fully delineate the complex and synergistic cognitive linkages between adolescent youths’ social networks and smoking behavior.
